# Effect of age on spatial memory performance in real museum vs. computer simulation

**DOI:** 10.1186/s12877-019-1167-2

**Published:** 2019-06-13

**Authors:** Maria Korman, Patrice L. Weiss, Michal Hochhauser, Rachel Kizony

**Affiliations:** 1 E.J. Safra Brain Research Center for the Study of Learning Disabilities, University of Haifa, 199 Aba Khoushy Ave. Mount Carmel, Haifa, Israel; 2 Department of Occupational Therapy, University of Haifa, Haifa, Israel; 3 Department of Occupational Therapy, Ariel University, Ariel, Israel; 4 Center of Advanced Technologies in Rehabilitation, Sheba Medical Center, Tel Hashomer, Israel

**Keywords:** Spatial working memory, Aging, Cognitive abilities, Real-world setting, Simulation

## Abstract

**Background:**

Healthy older adults frequently complain on difficulty in recalling the locations of objects of everyday use. Cognitive abilities decline with normal aging; inefficiencies of information processing, as well as deterioration of neuronal structures, may impede the performance of complex cognitive skills such as spatial memory. Extraneous, task-irrelevant cognitive load in real environments is usually high and might interfere with spatial memory abilities of older adults. The purpose of this study was to determine (1) the extent to which older adults maintain their cognitive capacity during a spatial memory task as compared to young adults and (2) whether this capacity is affected by performance of the task in a real environment setting where the cognitive demands are similar to a simulation, but the physical demands (navigating via walking versus via a mouse) vary.

**Methods:**

In the museum, participants physically moved between display stations to locate hidden tokens performing a task in which an ongoing representation of previous searches had to be remembered. A comparable task was implemented via mouse actions on a computer simulation. Seventeen healthy older (60–80 years) and twenty younger (20–45 years) adults performed both tasks in a counterbalanced order.

**Results:**

The younger group was superior to the older group in terms of success rate and completion time for both conditions. All participants performed better during the simulated task. The delta between the total performance score in the two settings of the older group was significantly larger as compared to the younger group, suggesting a differential impact of setting on the groups.

**Conclusions:**

Our results highlight the importance and feasibility of experimentation in ecologically relevant settings: differences were found in the way the cognitive performance of older and younger adults was affected by setting. Older adults appear to preserve basic cognitive abilities required for successful performance of object–location memory tasks. However, real museum setting appeared to impose higher demands on the older adults.

**Electronic supplementary material:**

The online version of this article (10.1186/s12877-019-1167-2) contains supplementary material, which is available to authorized users.

## Background

Spatial memory is a complex multidimensional process which includes a variety of components that help people to orient and act in space [1–3]. Spatial memory includes the ability to remember the spatial layout of environments, to know how to travel from one place to another, to remember the locations of objects within a specific environment, to have knowledge about the spatial arrangements of objects relative to each other, and to know one’s own location in the environment [3]. Performance in a spatial working memory task is dependent on the demands of active processing [4]. Object–location memory is a sub-type of spatial memory that requires working memory processing to link object identity information to location information [2, 5]. The processing demands of object-location memory are high since they require active manipulation of an object’s status information before the participant can respond [6].

Both cognitive and motor abilities decline with normal aging, impeding the performance of complex cognitive skills such as spatial memory [7–9]. Indeed, a frequent complaint of healthy older adults is their difficulty in recalling the locations of objects of everyday use [10]. Poorer object-location memory was demonstrated in older as compared to younger adults [11–13]. Learning about an environment, reflecting the efficiency of mental spatial representations (produced and memorized from listening to route descriptions) was shown to be age-dependent; middle-aged and older adults performed worse than young adults in a free mental recall task [14]. Furthermore, individual differences in working memory performance increase with age, as reflected by the larger variability of performance among the elderly [15–17].

In healthy aging, deficits in spatial tasks may be caused by inefficiencies of information processing, rather than by true deterioration of neuronal mechanisms or structures [18, 19]. The negative influence of advanced age on performance entailing spatial skills was shown to be dependent on the nature of the spatial task; spatial self-assessment of the sense of direction and orientation strategy is better preserved relative to performance in tasks demanding spatial visualization, mental rotation, and perspective taking [20]. Older adults may be primarily impaired in their ability to link contextual elements into a coherent episode and to make good use of attentional resources, such as the ability to sustain information processing over time, suppress irrelevant information or switch between activities [21, 22]. Moreover, older adults solved tasks of object–location memory, by additional recruitment of stimulus–response associations, which may provide partial compensation for their limited attentional resources [23].

Performance in ecological environments imposes increased perceptual and attentional loads due to the inherent sensory richness of such settings [24] - the extraneous cognitive load that does not contribute to the learning process itself in real environments is usually high and might interfere with spatial memory abilities of the elderly [19, 25, 26]. Nevertheless, many of the tests used to assess cognitive decline have consisted of artificial tasks that have little resemblance to the everyday activities. Using artificial tasks also implies that research participants may have little practice doing them, potentially contributing to deficits in performance, especially for those who are not able to adapt quickly (e.g., older adults). Such tasks may, therefore, not be good predictors of real-world cognitive behaviours [23, 27]. The testing of experimental paradigms under realistic conditions that include ecologically relevant cognitive load, gross motor movement and real interaction with objects, may not only lead to novel interventions for age-related cognitive impairments, but may also contribute to an improved understanding of the cognitive mechanisms in the mature brain.

The goal of the present study was to quantify the effect of both age and testing environments (real museum versus simulation of the museum) on object-location memory of healthy older and young adults. Two research questions were addressed. 1. To what extent do older adults maintain their cognitive capacity during a dynamic spatial working memory task as compared to young adults? 2. How is this capacity affected by performance of the task in a real environment setting where the cognitive demands are similar to a simulation, but the physical demands (navigating via walking versus via a mouse) vary?

In particular, we tested the following hypotheses:

1. Effect of the age group in both settings. Older adults will have a significantly weaker performance in both settings than younger adults as reflected by success rates, number of second attempts and performance speed.
2. 2a. Effect of setting on performance. Due to the higher cognitive and motor requirements of the real environment, both younger and older adults will have a significantly weaker performance in it compared to the simulated environment as reflected by success rates, number of second attempts and performance speed.
3. 2b. The deterioration in performance due to setting will be significantly greater for the older adults than for the younger adults.
4. The effect of difficulty level on performance within each setting. The performance of both younger and older adults in both settings will be significantly poorer as the level of difficulty increases (i.e., number of targets in a given attempt).
5. The effect of setting on the subjective experience: The older adults will rate the experimental task to be significantly more challenging than rated by the younger adults in both settings.

## Methods

### Participants

Twenty young healthy adults (9 male, 45%) aged between 22 and 35 years (27.4 ± 4.4) and 17 older community dwelling, healthy (6 male, 35%) adults aged between 60 and 79 years (70.10 ± 5.7) were recruited through advertisements. Pre-screening was done by a short telephone interview (including questions of general health status and basic demographic data) to exclude persons with a diagnosed neurological disease or orthopedic injury that affected walking. On the experimental day, participants completed several questionnaires to exclude those with cognitive, neurological, psychiatric or musculoskeletal dysfunctions, users of psychotropic medication and heavy alcohol consumers (more than 3 drinks per day). A Demographic & General health questionnaire was used to screen overall health and well-being SF-12 [28]. A small honorarium for participating in the study was payed. Ethical approval was obtained from the Institutional Review Board of the Faculty of Social Welfare and Health Sciences at the University of Haifa.

### Instruments

Demographic & General health questionnaire SF-12*.* These items documented participant gender, age, level of education, prior usage of simulation, general health and well-being.

#### Cognitive assessment

*Montreal Cognitive Assessment (MOCA)* was used to evaluate cognitive functioning and to screen participants with mild cognitive dysfunction. The total possible score is 30 points. The total possible score is 30 points. Participants were excluded if they scored less than 20 points [29]. The MOCA was introduced to both age groups to: 1) preserve similar experimental protocol and length between the groups; 2) to assess the possibility of relative differences in supra-threshold MOCA scores between the young and the elderly participants. Although the MOCA was originally developed to detect mild cognitive impairment in older adults, in the past 5–10 years it has also been widely used in research as well as in clinical settings as a screening tool for general cognitive abilities with younger adults as well [30, 31].

*SFQ* (Short Feedback Questionnaire) questionnaire [32] was used to obtain information related to the subjective experience of the participants. Participants rated their enjoyment and perception of success and control as well as perceived difficulty of the task in each setting (real and simulated environments) on a 5-point Likert scale (1 – very much to 5 – not at all). The participants were also asked to indicate their interest in the two tasks.

### Settings and tasks

The experiment was conducted in two settings. The Simulation setting took place in a small, quiet room with the participant seated at a computer facing a 15 in. screen and using a standard computer mouse to navigate and select items. The On-Site setting was located on the second floor of the Hecht archaeological museum which was instrumented with iPods placed at specific target locations. The total experimental time ranged from 75 to 90 min.

The task used in the study is a version of the ‘traveling salesman problem’, so called because it refers to the difficulty that traveling salesmen have in remembering which houses they have already visited and in which of those houses they have made a successful sale. This problem was transformed into spatial search task that assessed the ability to retain and manipulate information in spatial working memory. Previous research has demonstrated that the performance in this task reliably discriminated between different clinical populations, e.g., patients with frontal lobe excisions, temporal lobe excisions or amygdalo-hippocampectomy and healthy controls [33, 34]. Difficulties in performing this task were previously shown to be related to poor ‘strategic’ or ‘executive’ functions in frontal lobe patients [33, 34]. In contrast, successful performance required a self-ordered, well-organized search, which presumably reflects both spatial memory capacity and the effectiveness of dynamic update of target value [33, 35].

**Fig. 1:**
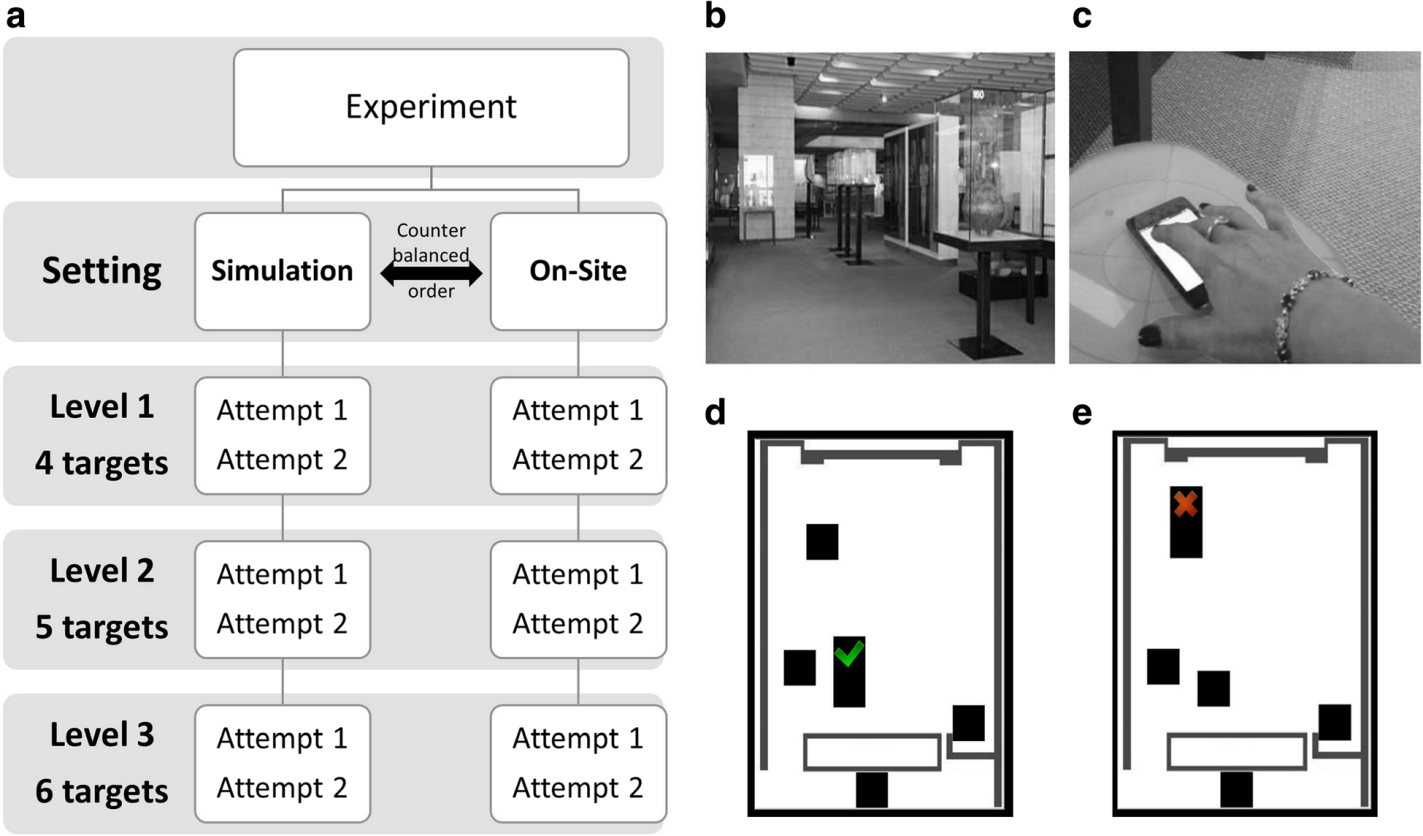
**a** Experimental procedure; **b** On-Site (museum) setting - experimental area in the museum (left); **c** Participant’s hand selecting a target while searching for a token (right); **d** Simulation setting screen examples. Five targets with one of the targets revealing a token (green “checkmark” symbol above the target), when the participant clicked on this target; **e** Five targets with one of the targets revealing a “X” symbol, when the participant made an error

All participants performed the spatial challenge in two settings: the computer simulation (Simulation) and the museum setting (On-Site), order of the first setting was counterbalanced. In both settings, successful completion of the task requires that the participant maintain and update an ongoing representation of previous searches in different locations (targets) and develop an appropriate search strategy (Fig. 1). The task required the participants to search a set of targets (4, 5 or 6 targets, corresponding to difficulty levels 1–3) by “revealing” them (i.e., by clicking on the mouse button in the Simulation setting or touching the iPod target icon with a finger in the On-Site setting, see videos (Additional files 1-3). A click or touch caused the target to reveal whether it was empty or contained a token (green “V” marks or empty squares were shown while the target was pressed). Two attempts were allowed for each difficulty level.

At each difficulty level, a continuous search for dynamically hidden tokens was performed. During any given search, a single token was hidden at only one of the target locations. The participants began the attempt by opening the targets in any order until they located the first token. Once a token was found within a given target, that target could not be used to hide another token until the end of that attempt. The token was re-hidden in one of the remaining targets and searched for again until 4, 5 or 6 tokens were found (depending on the level). The participants were instructed that any target that had already been visited should not be searched again until the end of that attempt. This requirement meant that they had to remember which targets had been searched and found to be empty (during the current search) or contained a token (during previous searches in that attempt). The number of tokens was equal to the number of targets, but the order of hiding the tokens was different across the attempts. Performance errors occurred when a user returned to a previously searched target that either contained a revealed token or was empty. Any error aborted the current attempt. The task ended successfully when the participant found all the tokens (in each of the targets in the current layout).

Graphic display of the target content was similar in both settings. Empty targets were designated by an empty black frame shown above the target, errors were designated by a red “X” shown above the target (Fig. 1e) and a found token was designated by a green “*checkmark*” (Fig. 1d). The salient instruction was that once a token had been found at a particular target, the target should not be searched for again until the end of the attempt (within search error). All attempts started from the Start target, located in the same place in all layouts. Start target disappeared after the search was initiated.

#### On-site museum setting

A 40 by 40 m section of the museum was used (Fig. 1b). Participants were presented with targets on a touch-sensitive iPod screen, with each iPod placed on a stand at specific locations in the museum space (Fig. 1c). While the location of the iPods in the museum was real and constant, the tokens were virtual (i.e., they appeared on the iPod screen when touched by the participant). iPod locations were spaced 2 to 30 m from each other. Only one target (black square) was displayed on each iPod. Locations of all iPods for a given round were shown to the participant prior to the commencement of each attempt to ensure that the layout of targets was obvious (as occurred in the Simulation setting). Thus, participants were not required to look for the iPods, only to physically approach them and check their content. Participants were required to search for the targets until they revealed a green “checkmark” token when touched. A videotaped example of performing in the On-Site setting is shown in Additional file 1.


Additional file 1:A videotaped example of performing in the On-Site setting: Level 1, 4 targets. The first and the second tokens are successfully found. During the search for the third token an error is made. This attempt is invalid. (WMV 5120 kb)


#### Simulation setting

Participants were presented on a computer screen with targets that were distributed on an outline that resembled the museum setting (Fig. 1d and e). The search for targets was performed by navigating with a standard computer mouse and clicking the left button upon reaching the target. Examples of a 5-target round are found in Additional file 2 and Additional file 3. These video recordings demonstrate how different search strategies can lead to a successful performance.


Additional file 2:A videotaped example #1 of performing in the Simulation setting: Level 2, 5 targets. All tokens are successfully found. This attempt is valid. Note the order of the targets are opened. (WMV 1880 kb)



Additional file 3:A videotaped example #2 of performing in the Simulation setting: Level 2, 5 targets. All tokens are successfully found. This attempt is valid. Note that the targets are opened in a different order. (WMV 2100 kb)


### Procedures

Each participant participated in a single 60–75 min session which started by signing an informed consent after receiving an explanation of the experiment. Participants were familiarized with the tasks using only three targets until they successfully performed three successive training trials in each setting. All participants performed in both settings in a counter-balanced order with a 15 min break between settings. They completed the usability questionnaires at the conclusion of each experimental task. In each setting, the participant performed three rounds of increasing difficulty: a first round of four target locations, a second round of five target locations and a third round of six target locations. Target locations were pseudo-randomly arranged for each round to prevent learning of spatial location and possible transfer of knowledge between attempts and settings. In the case of an error, the current round was aborted and the participant was given a second attempt to complete it at the same difficulty level (but with a different spatial arrangement of targets). Thus, depending on the participant’s success, the maximum number of trials in each setting was six (two at each difficulty level) and the minimum number was three.

### Data analysis

A *frequency of success* score was tabulated as the number of participants who successfully completed the attempts in their first or second attempt. A *performance* score was calculated for successfully competed attempts as the time to complete the attempt divided by the number of clicks made by the participant during the attempt. The Performance score was calculated only for successfully completed attempts (either the first or the second attempt in any given level). As the number of clicks depended on the individual search strategy, the Performance score provides a measure of per click planning plus execution time normalized by the number of clicks.

The data were analyzed using the IBM SPSS Statistics Version 21.0 software. ANOVA repeated measures mixed design model with one within-subjects factor (setting) and one between-subjects factor (age group) was performed to compare total performance scores (mean performance score of the three difficulty levels) across settings and groups. Since some data were not normally distributed, non-parametric Mann-Whitney tests were used to compare performance on setting and difficulty level between groups. The SFQ data were analyzed using Mann-Whitney U-tests to compare between feedback from the younger and older groups.

## Results

### Between group comparisons

The young group had a higher MOCA score (median = 28 IQR = 25–29; M = 27.1 SD = 2.3) than the older group (median = 26 IQR = 23.5–28; M = 25.7 SD = 2.6), these differences were not significant (u = 119.5; *p* = 0.07). Given their high MOCA values, the older adults in our sample showed no evidence of an undiagnosed mild cognitive impairment.

**Fig. 2:**
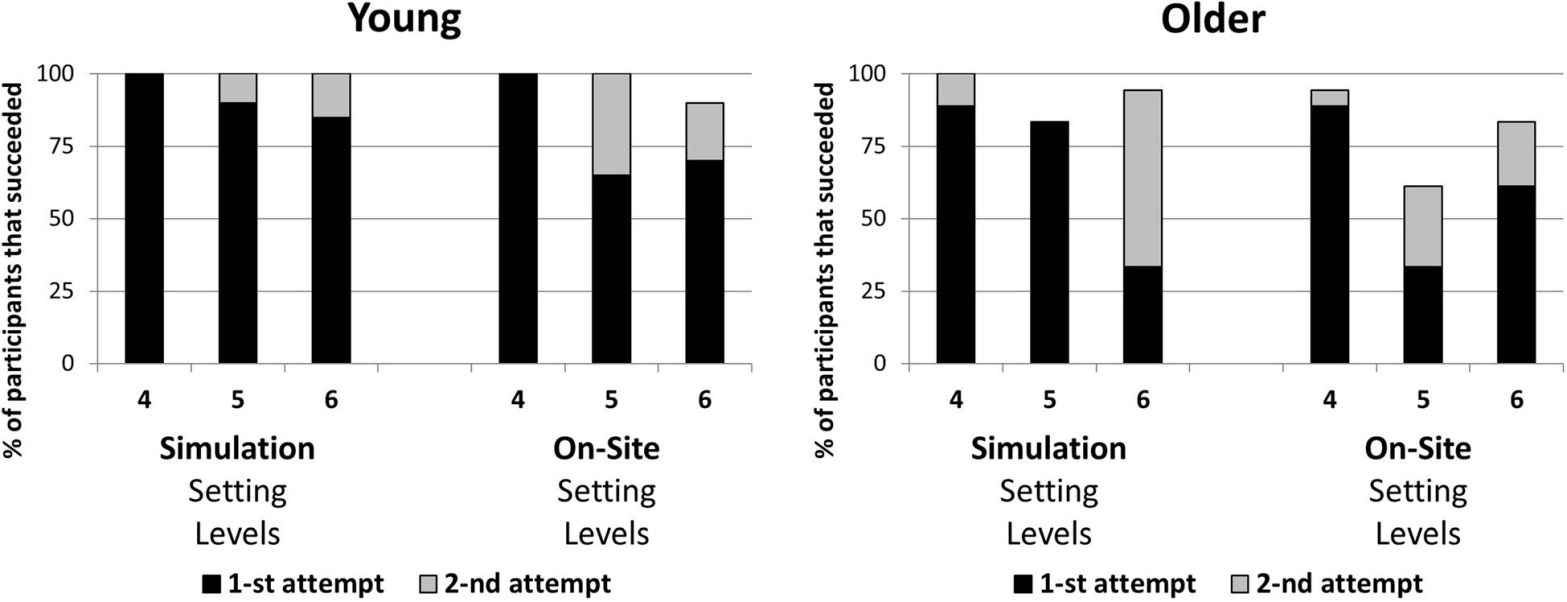
Percentage of participants that completed a given difficulty level (4, 5 or 6 targets) in a given setting (On-Site or Simulation). Black histograms denote the first attempt, grey histograms denote the second attempt

The frequency of success and highest achieved level of difficulty were dependent on age and the number of attempts (Fig. 2). In the Simulation setting, 100% of the young participants successfully completed the trials at all difficulty levels, with the vast majority succeeding at the first attempt (black). In contrast, although all the older adults successfully completed the easiest level, only 75–85% succeeded at the two more difficult levels. At the third difficulty level, the majority (60%) succeeded only from the second attempt (grey).

In the On-Site setting, all of young adults succeeded at the easiest and the middle difficulty levels and 90% at the third difficulty level. However, in contrast to the Simulation setting, at the second and third difficulty levels, a second attempt was needed by about 30%. The older adults were less successful at all levels, with only 61% success rate at the middle level and 83% at the third difficulty level, with more than 28 and 23% respectively, succeeding only at their second attempt.

Calculating the frequency of success at the first trial across all difficulty levels showed that in the On-Site setting there were no significant differences between the groups (χ^2^_(2)_ = 4.5; NS). Forty percent of the younger adults succeeded in the first trial at all three difficulty levels, 55% at two difficulty levels and 5% at only one difficulty level; 25 % of older adults succeeded from the first trial at all difficulty levels, 43.8% at two difficulty levels and 31.3% at only one difficulty level. In contrast, in the Simulation setting, the difference between the frequency of success between the age groups was significant (χ^2^_(2)_ = 8.0; *p* = .018). Seventy-five percent of the younger adults succeeded in the first trial at all difficulty levels and 25% at two difficulty levels whereas only 35.3% of the older adults succeeded in the first trial at all difficulty levels, 41.2% at two difficulty levels and 23.5% at only one difficulty level.

The mean time to successfully complete the task was always slower for the older group compared to the young group in both settings and across all difficulty levels (see Table 1). However, as the number of clicks per difficulty level depended on the individual search strategy (see Methods), the statistical analysis was performed using a normalized performance score calculated as the performance time normalized by the number of clicks per level.

**Table 1:** Mean time to successfully complete a given level in both settings

		Mean (s)	Std. Deviation
Young	Four targets Simulation	8.6	3.4
	Five targets Simulation	14.3	4.6
	Six targets Simulation	17.9	7.1
	Four targets On-Site	64.7	19.2
	Five targets On-Site	82.9	23.4
	Six targets On-Site	100.9	20.2
Older	Four targets Simulation	16.7	7.9
	Five targets Simulation	21.2	3.9
	Six targets Simulation	22.5	5.6
	Four targets On-Site	91.4	48.9
	Five targets On-Site	143.5	93.5
	Six targets On-Site	154.1	77.7

The younger participants had significantly lower performance scores than the older participants in all difficulty levels in both settings, showing that the “per click” time of the younger participants was faster (Table 2).

**Table 2:** Between group comparisons of performance scores (seconds/number of clicks) according to difficulty levels and settings. Note that number of participants analyzed at each difficulty level and setting is different since only successfully completed trials were included

Condition/setting	Young			Older			Mann Whitney
	*N*	Median (IQR)	Mean (SD)	*N*	Median (IQR)	Mean (SD)	U (p)
On-Site setting							
Four targets	20	7.13 (6.38–8.2)	7.76 (1.99)	16	11.38 (9.46–13.64)	12.81 (6.98)	36.5 (.0001)
Five targets	20	7.43 (6.78–9.28)	7.96 (1.63)	10	13.11 (9.63–17.96)	14.85 (7.01)	18.0 (.0001)
Six targets	18	7.52 (6.63–8.41)	7.65 (1.26)	13	10.27 (9.15–16.68)	13.17 (6.22)	22.0 (.0001)
Mean of successful trials		7.48 (6.88–8.66)	7.79 (1.26)		11.64 (9.78–15.92)	12.94 (4.78)	26.0 (.0001)
Simulation setting							
Four targets	20	1.33 (1.09–1.67)	1.40 (0.38)	17	2.35 (1.83–3.41)	2.65 (1.11)	27.0 (.0001)
Five targets	20	1.23 (1.07–1.70)	1.38 (0.53)	14	1.94 (1.71–2.37)	2.11 (0.63)	41.0 (.001)
Six targets	20	1.28 (1.04–1.69)	1.40 (0.49)	13	1.93 (1.71–2.41)	2.05 (0.43)	36.0 (.001)
Mean of successful trials		1.20 (1.12–1.57)	1.39 (0.43)		2.23 (1.89–2.71)	2.31 (0.57)	30.0 (.0001)

### Within group comparisons and interactions

**Fig. 3:**
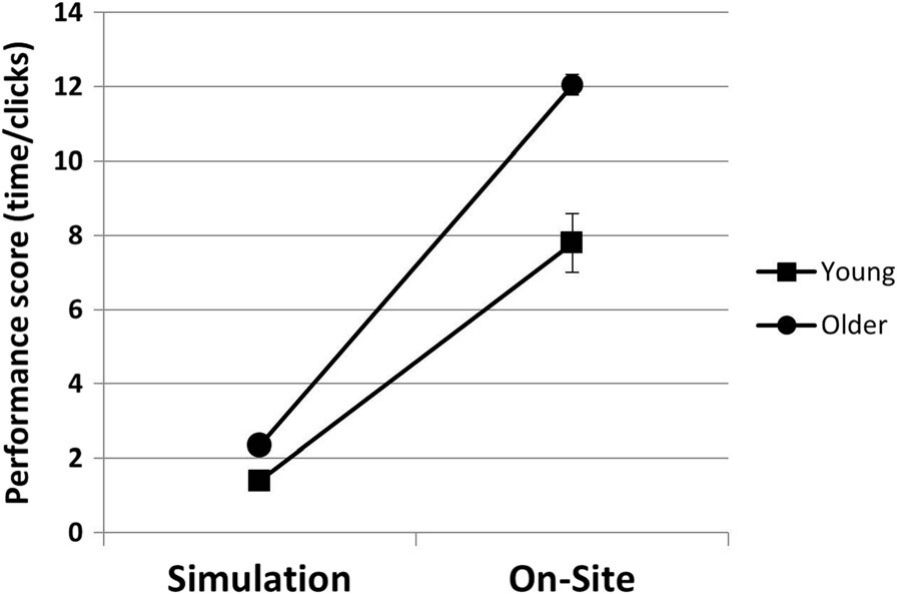
Impact of setting (On-Site, Simulation) on the mean total performance score according to the age group (Young, Older). Note the interaction effect between setting and age. Positive values indicate that performance scores are slower. Bars – STD

The total performance scores (mean performance score of the three difficulty levels) in the Simulation setting was significantly greater than in the On-Site setting for both the young and older adults, with younger adults outperforming their older peers. An ANOVA showed a significant main effect for setting (F(1,35) = 221.79; *p* = .0001; ƞ2p = .86) and for age (F(1,35) = 30.73; p = .0001; ƞ2p = .47) with large effect sizes. In addition, a significant interaction effect between age groups and settings with a medium effect size was found (F(1,35) = 13.66; *p* = .001; ƞ2p = .28). Post-hoc t-test analysis showed that the interaction was due to significant between groups differences for both settings, On-Site (t(18) = − 4.31, p = .0001) and Simulation (t(35) = − 5.59, *p* = .0001). Figure 3 presents the performance scores of each group in both settings.

To further assess the impact of setting for each age group, the delta between total performance score values in the On-Site setting and in the Simulation setting was calculated for each participant. The mean delta of the older group was significantly larger (mean ± SD = 10.62 ± 4.95) as compared to the younger group (mean ± SD = 6.40 ± 1.23; t(18) = − 3.43, *p* = .003), suggesting a differential impact of setting on the groups.

No correlations were found between the mean total performance scores in the On-Site and Simulation setting for either age group, suggesting that the two tasks, each in a different setting, seem not to share the same cognitive processes, irrespective of age (Pearson correlations: Young, r = 0.23, *p* = 0.351; Older r = − 0.245, *p* = 0.3281). Also the correlations between the mean performance scores at each of the difficulty levels in the On-Site and Simulations settings were non-significant.

### Usability

Analysis of subjective experiences of the participants (scores varied from 1 = not at all to 5 = very much) showed that the older participants found the task to be significantly more difficult than young participants in both settings (On-Site mean ± SD scores: 4.6 ± 0.7 (older) vs. 2.9 ± 1.2 (younger), U = 53.0, *p* < 0.001 and Simulation mean scores: 4.4 ± 0.8 (older) vs. 3.2 ± 0.8 (younger), U = 40.5, p < 0.001). The older participants were also more interested than younger adults in the task in both settings (On-Site mean scores: 1.5 ± 0.6 vs. 2.1 ± 0.9, U = 110, *p* = 0.048 and Simulation mean scores 1.7 ± 0.6 vs. 2.5 ± 0.9, U = 81, *p* = 0.004). There were no significant differences between the two age groups in the levels of enjoyment of and satisfaction with the task in either setting (both were high, mean scores < 2).

## Discussion

The current study allowed direct comparison of performance in object-localization memory task between simulated and physical settings in healthy older and young adults. For both groups it took longer to complete the On-Site task due to physical distances between targets. Attention and working memory had to be recruited for a longer time, which might have been more difficult for the older adults [21, 22], especially when maintenance rehearsal [36] of the items in the working memory is needed during longer trials.

Older adults appear to preserve basic cognitive abilities required for successful performance of object–location memory tasks. At the easiest levels of difficulty in both settings, both groups had no deficits in the frequency of success to complete the level, i.e., they coped well with the demands of the “Travelling salesman” task. However, at the higher levels of difficulty, an increasing gap in performance between the two groups became evident, with relative losses ranging from 20 to 40% in the frequency of success for both settings. Thus, the negative effect of advanced age on performance was exacerbated by a greater number of targets. This result is in contrast to the recent finding of Bennett, Loomis, Klatzky, & Giudice (2017) that showed no interaction between age and memory load in a task that required mentally keeping track of the directions and distances of previously viewed objects in a real-world environment. Older adults were also consistently slower in in both settings. These findings are compatible with numerous studies that show a generally lower capacity of working memory [37], long-term spatial memory [38] and slower processing speed (e.g., [21]) in older adults. Interestingly, a previous study comparing active vs. passive motor exploration during route learning suggested that active learning with a joystick was detrimental to navigational performance as compared to passive learning (from observation without movements) for both young and older adults [39]. This finding was interpreted as an outcome of dual-tasking; active learning demanded a division of cognitive resources between operating the input device and implementing directional instructions. In the current study, both settings required active exploration and movement; in the Simulation setting it entailed fine motor control of the computer mouse and in the On-Site setting it entailed gross motor (walking) and fine motor (interaction with iPads) movements. Additional studies are needed to evaluate the contribution of the motor activity component to differences obtained in performance of the same task in a real versus a simulated environment.

Older adults had larger losses in performance in the On-Site setting as compared to young participants, perhaps related to the greater frequency of task-extraneous stimuli which presumably impose a higher cognitive load [24]. Distraction in real world settings, such as driving [40] and shopping [41] can be difficult to control. This finding supports previous studies showing that older adults appear to have difficulty in maintenance of focus on the goal in the face of distraction, in their allocation of attentional resources, and in their ability to engage in complex activities and task switching [21, 22, 42]. Moreover, the demands of active movement may add to the difficulty for the older group due to age-related declines in functional capacity and muscle strength and mass and deterioration of gait [43, 44]. The difference in speed of navigating the On-Site route between older and younger adults could thus also contribute to the observed discrepancies in the cognitive performance. Our interpretation of the findings is limited by the fact that we did not use additional measures to assess spatial working memory, attentional control, processing speed and motor functioning, due to the length of the experiment (up to 90 min). In future studies it is recommended to add such outcomes to control for their effects on performance.

Analysis of subjective attitudes towards the two experimental settings revealed that while all participants had a positive user experience (i.e., they were interested in and were satisfied with both tasks), the older adults found the object-localization memory task to be significantly more challenging than the young participants; this difference is compatible with the behavioral results. Nevertheless, the On-Site setting was reported to be a more satisfying user experience by both age groups. This observation suggests that ecological versions of common laboratory tasks, such as cognitive tests, are not only feasible, but also attractive for the participants; both young and older adults tend to respond positively to virtual games and simulated tasks as do many rehabilitation populations (e.g., [45]).

Functional activity in real life situations is one of the most important outcomes to quantify [46]. Cognitive and meta-cognitive abilities’ evaluation using ecologically valid assessments has been discussed extensively (e.g., Burgess et al., 2006; Katz & Maeir, 2011; Parsons et al., 2017). The most valid way to do this would appear to be to assess performance of a complex task such as shopping in the real world, as suggested by Shallice and Burgess (1991) who developed the Multiple Errands Test (MET) and by Hamera and Brown (2000) who developed the Test of Grocery Shopping Skills. However, this type of test is time consuming, requires special settings and often a budget for each administration (Nir-Hadad et al., 2017). The use of simulations or virtual reality-based tests has been suggested as a solution for these challenges as they enable the use of settings that require similar skills as in the real world (Kizony, 2018; Weiss et al., 2014; Rand et al., 2007). The results of the current study are in contrast with studies that reported similarities between performance of a functional-cognitive test in the simulation compared to the real world (e.g., Rand et al., 2009; Nir-Hadad et al., 2017; Renison, Ponsford, Testa, Richardson, & Brownfield, 2012), and a high level of enjoyment from the simulation (e.g., Rand et al., 2009; Nir-Hadad et al., 2017). This discrepancy may be due to the relative simplicity of the display of the simulation used in the current study as well as the additional motor load in the On-site setting. Nevertheless, the current results highlight the continuing need to query the extent to which simulations resemble real life performance in both objective and subjective aspects, and to determine their dependence on environmental and personal factors.

Currently, there are only a few studies that have addressed age-related differences in navigational abilities and spatial memory performance in a real versus a simulated setting [47–49]. Our results support their overarching conclusion highlighting the importance of experimentation in ecologically relevant settings. Especially, given the differences found in the way the real setting affected the cognitive performance of older and younger adults. Such differences are expected to be even more apparent in clinical populations. A deeper understanding of age- and medical condition-induced constraints on spatial working memory management may help in making an optimal use of the available cognitive processing capacity.

## Conclusion

Our results highlight the feasibility of experimentation in ecologically relevant settings as well as the clinical potential of the ensuing results. Differences were found in the cognitive performance of older and younger adults in the two settings. There is a continuing need to query the extent to which performance in simulated settings resembles real life performance in both objective and subjective aspects, and to determine their dependence on environmental and personal factors.

## Data Availability

The datasets used and/or analysed during the current study are available from the corresponding author on reasonable request.

## Abbreviations

ANOVA: Analysis of variance
MOCA: Montreal Cognitive Assessment
SFQ: Short Feedback Questionnaire

## References

[1] Kessels RP, de Haan EH, Kappelle LJ, Postma A (2001). Varieties of human spatial memory: a meta-analysis on the effects of hippocampal lesions. Brain Res Brain Res Rev.

[2] McAfoose J, Baune BT (2009). Exploring visual–spatial working memory: a critical review of concepts and models. Neuropsychol Rev.

[3] Postma A, Kessels RP, van Asselen M (2008). How the brain remembers and forgets where things are: the neurocognition of object-location memory. Neurosci Biobehav Rev.

[4] Cornoldi C, Vecchi T (2004). Visuo-spatial working memory and individual differences. Appl Cogn Psychol.

[5] King JA, Trinkler I, Hartley T, Vargha-Khadem F, Burgess N (2004). The hippocampal role in spatial memory and the familiarity--recollection distinction: a case study. Neuropsychology.

[6] Logie RH (2011). The functional organization and capacity limits of working memory. Curr Dir Psychol Sci.

[7] Salthouse TA (2010). Selective review of cognitive aging. J Int Neuropsychol Soc.

[8] Salthouse TA (2017). Shared and unique influences on age-related cognitive change. Neuropsychology.

[9] Moffat SD (2009). Aging and spatial navigation: what do we know and where do we go?. Neuropsychol Rev.

[10] Bolla KI, Lindgren KN, Bonaccorsy C, Bleecker ML (1991). Memory complaints in older adults. Fact or fiction?. Arch Neurol.

[11] Mitchell KJ, Johnson MK, Raye CL, Mather M, D'Esposito M (2000). Aging and reflective processes of working memory: binding and test load deficits. Psychol Aging.

[12] Kessels RP, Hobbel D, Postma A (2007). Aging, context memory and binding: a comparison of "what, where and when" in young and older adults. Int J Neurosci.

[13] Cheke LG (2016). What-where-when memory and encoding strategies in healthy aging. Learn Mem.

[14] Meneghetti C, Borella E, Muffato V, Pazzaglia F, De Beni R (2014). Environment Learning from Spatial Descriptions: The Role of Perspective and Spatial Abilities in Young and Older Adults.

[15] Nagel IE, Chicherio C, Li SC, von Oertzen T, Sander T, Villringer A, Heekeren HR, Backman L, Lindenberger U. Human aging magnifies genetic effects on executive functioning and working memory. Front Hum Neurosci. 2008;2(1). 10.3389/neuro.09.001.2008 PMC2525971 18958202

[16] Soei E, Daum I (2008). Course of relational and non-relational recognition memory across the adult lifespan. Learn Mem.

[17] Uttl B, Graf P (1993). Episodic spatial memory in adulthood. Psychol Aging.

[18] Shih S-I, Meadmore KL, Liversedge SP (2012). Aging, eye movements, and object-location memory. PLoS One.

[19] Peleg-Adler R, Lanir J, Korman M (2018). The effects of aging on the use of handheld augmented reality in a route planning task. Comput Hum Behav.

[20] Borella E, Meneghetti C, Ronconi L, De Beni R (2014). Spatial abilities across the adult life span. Dev Psychol.

[21] Coubard OA, Ferrufino L, Boura M, Gripon A, Renaud M, Bherer L (2011). Attentional control in normal aging and Alzheimer's disease. Neuropsychology.

[22] Deary IJ, Corley J, Gow AJ, Harris SE, Houlihan LM, Marioni RE, Penke L, Rafnsson SB, Starr JM (2009). Age-associated cognitive decline. Br Med Bull.

[23] Meulenbroek O, Kessels RP, de Rover M, Petersson KM, Rikkert MG, Rijpkema M, Fernandez G (2010). Age-effects on associative object-location memory. Brain Res.

[24] Sweller J (1994). Cognitive load theory, learning difficulty, and instructional design. Learn Instr.

[25] Lorsbach TC, Simpson GB (1988). Dual-task performance as a function of adult age and task complexity. Psychol Aging.

[26] Van Gerven PWM, Paas FGWC, Van Merriënboer JJG, Schmidt HG (2002). Cognitive load theory and aging: effects of worked examples on training efficiency. Learn Instr.

[27] Korman WPL, Kizony R (2016). Living labs: overview of ecological approaches for health promotion and rehabilitation. Disabil Rehabil.

[28] Ware J, Kosinski M, Keller SD (1996). A 12-item short-form health survey: construction of scales and preliminary tests of reliability and validity. Med Care.

[29] Nasreddine ZS, Phillips NA, Bedirian V, Charbonneau S, Whitehead V, Collin I, Cummings JL, Chertkow H (2005). The Montreal cognitive assessment, MoCA: a brief screening tool for mild cognitive impairment. J Am Geriatr Soc.

[30] Kousaie S, Sheppard C, Lemieux M, Monetta L, Taler V. Executive function and bilingualism in young and older adults. Front Behav Neurosci. 2014;8(250). 10.3389/fnbeh.2014.00250 PMC4111127 25120442

[31] Bernard JA, Seidler RD (2013). Relationships between regional cerebellar volume and sensorimotor and cognitive function in young and older adults. Cerebellum.

[32] Kizony R, Kaz N, Rand D, Weiss T (2006). Short feedback questionnaire (SFQ) to enhance client-centered participation in virtual environments. CyberPsychol Behav.

[33] Owen AM, Morris RG, Sahakian BJ, Polkey CE, Robbins TW (1996). Double dissociations of memory and executive functions in working memory tasks following frontal lobe excisions, temporal lobe excisions or amygdalo-hippocampectomy in man. Brain.

[34] Owen AM, Downes JJ, Sahakian BJ, Polkey CE, Robbins TW (1990). Planning and spatial working memory following frontal lobe lesions in man. Neuropsychologia.

[35] Korman, Kizony R, Hochhauser M, Kuflik T, Wecker A, Weiss P. Spatial working memory performance in real museum environment versus computer simulation: a comparison between healthy elderly and young adults. Proc 10th Intl Conf Disability, Virtual Reality & Associated Technologies. Gothenburg: University of Reading's institutional Research Repository, CentAUR; 2–4 sept 2014 ISBN 978–0–7049-1546-6; 2014.

[36] Davelaar EJ (2013). Short-term memory as a working memory control process. Front Psychol.

[37] Jenkins L, Myerson J, Joerding JA, Hale S (2000). Converging evidence that visuospatial cognition is more age-sensitive than verbal cognition. Psychol Aging.

[38] Barnes CA (1988). Aging and the physiology of spatial memory. Neurobiol Aging.

[39] Taillade Mathieu, Sauzéon Hélène, Arvind Pala Prashant, Déjos Marie, Larrue Florian, Gross Christian, N’Kaoua Bernard (2013). Age-Related Wayfinding Differences in Real Large-Scale Environments: Detrimental Motor Control Effects during Spatial Learning Are Mediated by Executive Decline?. PLoS ONE.

[40] Li N, Jain JJ, Busso C (2013). Modeling of driver behavior in real world scenarios using multiple noninvasive sensors. Trans Multi.

[41] Nir-Hadad SY, Weiss PL, Waizman A, Schwartz N, Kizony R (2017). A virtual shopping task for the assessment of executive functions: validity for people with stroke. Neuropsychol Rehabil.

[42] Verhaeghen P, Cerella J (2002). Aging, executive control, and attention: a review of meta-analyses. Neurosci Biobehav Rev.

[43] Bauman Adrian, Merom Dafna, Bull Fiona C., Buchner David M., Fiatarone Singh Maria A. (2016). Updating the Evidence for Physical Activity: Summative Reviews of the Epidemiological Evidence, Prevalence, and Interventions to Promote “Active Aging”. The Gerontologist.

[44] Rosso AL, Studenski SA, Chen WG, Aizenstein HJ, Alexander NB, Bennett DA, Black SE, Camicioli R, Carlson MC, Ferrucci L (2013). Aging, the central nervous system, and mobility. J Gerontol A Biol Sci Med Sci.

[45] Weiss PL, Kizony K, Feintuch U, Rand K, Katz N. Virtual reality applications in neurorehabilitation. In: LC MES, Gage FH, Clarke S, Duncan PW, editors. Textbook of Neural Repair and Neurorehabilitation, vol. 2. Cambridge: Cambridge University press; 2014. p. 98–208.

[46] American Occupational Therapy A: Occupational therapy practice framework : domain & process. Bethesda, MD: AOTA Press/American Occupational Therapy Association; 2014.

[47] Kalova E, Vlcek K, Jarolimova E, Bures J (2005). Allothetic orientation and sequential ordering of places is impaired in early stages of Alzheimer's disease: corresponding results in real space tests and computer tests. Behav Brain Res.

[48] Cushman LA, Stein K, Duffy CJ (2008). Detecting navigational deficits in cognitive aging and Alzheimer disease using virtual reality. Neurology.

[49] Taillade M, N'Kaoua B, Sauzéon H. Age-related differences and cognitive correlates of self-reported and direct navigation performance: the effect of real and virtual test conditions manipulation. Front Psychol. 2016;6(2034). 10.3389/fpsyg.2015.02034 PMC4725096 26834666

